# Editorial: Brain-Behaviour Interfaces in Linguistic Communication

**DOI:** 10.3389/fnhum.2020.00324

**Published:** 2020-09-11

**Authors:** Olga Shcherbakova, Andriy Myachykov, Beatriz Martín-Luengo, Yury Shtyrov

**Affiliations:** ^1^Department of General Psychology, Faculty of Psychology, Saint Petersburg State University, Saint Petersburg, Russia; ^2^Laboratory of Behavioural Neurodynamics, Saint Petersburg State University, Saint Petersburg, Russia; ^3^Department of Psychology, Northumbria University, Newcastle upon Tyne, United Kingdom; ^4^Centre for Cognition and Decision Making, National Research University Higher School of Economics, Moscow, Russia; ^5^Department of Clinical Medicine, Center of Functionally Integrative Neuroscience, Aarhus University, Aarhus, Denmark

**Keywords:** brain, language, EEG, neurolinguistic, psycholinguistic, embodied cognition

Language is a uniquely human cognitive function, which greatly defines and determines our psychological and social traits. Despite the importance of language and speech, they remain among the least understood human cognitive processes, and their neurobiological underpinnings are still poorly understood.

In recent decades, an immense body of diverse data illuminating the neural bases of language processes in both children and adults has been acquired through the use of many advanced techniques. These include electroencephalography (EEG), magnetoencephalography (MEG), functional magnetic-resonance imaging (fMRI), transcranial magnetic stimulation (TMS), transcranial direct and alternating current stimulation (tDCS, tACS), eye-tracking, behavioral measures, etc. The combined power of these techniques continues to shed light upon the brain mechanisms of language acquisition, comprehension and processing, speech disorders, their diagnosis and treatment, as well as the interplay between language and other neurocognitive systems and functions.

The aim of the Research Topic *Brain-Behavior Interfaces in Linguistic Communication* is to provide a state-of-the-art overview of this diverse and multidisciplinary area of research, with a special emphasis on bridging the gap between different research fields, theoretical views, and methodologies.

Our Research Topic offers a collection of 14 articles on various facets of linguistic behavior and its neural underpinnings. The collection comprises 11 research papers (including six original research reports and five brief research reports), one comprehensive review, one mini review, and one opinion paper.

The collection can be topically divided into several groups of papers. The first group brings together several articles using electroencephalography in order to investigate *the neural bases* of language learning and use. The opinion article by Shtyrov et al. addresses the effectiveness and neural underpinning of two main routes of *novel word acquisition*: (1) explicit encoding and (2) implicit learning (fast mapping). The authors discuss methodological confounds besetting existing research paradigms and provide a clear perspective for designing a comprehensive and fully balanced experimental approach for comparing these two language learning modes. The experimental study described by Vasilyeva et al. follows up on this and investigates the neural bases of *fast mapping* in adults by documenting near-instant changes in neural activity after a single-shot novel word training. The authors conclude that fast mapping may promote rapid integration of newly learned items into the brain's neural lexicon, even into adulthood. In a related article on *ERP correlates of novel word learning*, Bermúdez-Margaretto et al. show how novel words repeatedly associated with meaningful cues demonstrate a higher attenuation of N400 responses than the words trained in a basic orthographic condition, confirming facilitation of the lexico-semantic processing of these stimuli as a consequence of semantic association. This finding suggests that novel word learning could be influenced by the activation of the categorization-related network. Next, the contribution by Ovchinnikova et al. investigated auditory event-related potentials in children reared in two very different types of environment: biological-family care or institutional care. The paper makes an important contribution concerning *the role of social environment in neurocognitive maturation*. den Hollander et al. further inform this debate by using EEG for identifying *the speech production stages* in early and late adulthood. They report no scalp distribution differences between the two groups suggesting that the same networks are involved at different stages, regardless of the age, even though the timing of the individual stages is different between the groups. Alday and Kretzschmar used ERP and multiple-response speed-accuracy trade-off (SAT) paradigm to investigate *the relationship between N400 and P300 ERP components*. The article clarifies how these two classic ERP potentials determine behavioral profiles. With the use of multivariate Bayesian mixed-effects models, GLMM-based approach, and partial effects, the paper demonstrates how overlapping ERP responses in one sample of participants predict behavioral SAT profiles of another sample. Moreover, this research confirms that the P300 and N400 reflect two independent but interacting processes and that the competition between these processes is reflected differently in the speed-accuracy trade-off behavior. Finally, in an EEG study on *a language in transition* (Icelandic) Bornkessel-Schlesewsky et al. show that the neurophysiological responses already reflect projected language changes that are not yet apparent in the overt behavior of native speakers.

Another set of articles address *semantic aspects* of language learning and use. The mini review by Mkrtychian et al. offers a snapshot of psycholinguistic and neurocognitive approaches to studying *concrete and abstract semantics*. A review by Monaco et al. discusses the role of *embodied semantics in second language comprehension* arguing that L2 is embodied differently than L1 (which might have important clinical implications). Lastly, the research by Calabria et al. addresses the issue of *semantic processing in bilingual* (Catalan—Spanish) *aphasia*. The results suggest that lexical retrieval in individuals with bilingual aphasia may be selectively impaired within their non-dominant language due to an excessive amount of inhibition placed upon this language.

Two contributions from our collection focus on investigating *reading processes* using *eye-tracking*. Lou et al. suggest that eye movements during reading can be influenced by the *motivation of self-enhancement* in addition to various stimulus' properties and cognitive factors; this also indicates that eye-tracking can be used to study implicit social cognition. Research presented by Petrova et al. shows that *readers process information better and faster while reading sketch-notes than verbal texts*; additionally, various types of sketch-notes differ in terms of how good the readers are in following the order of elements.

Finally, two articles offer examples of *behavioral psycholinguistic* research. Niebuhr et al. report the results of a 12-weeks *prosodic charisma training* that is shown to be more beneficial for female speakers as opposed to male ones. Pokhoday et al. report new evidence about the role of *the speaker's attention* (manipulated by visual priming) and event orientation in sentence production by using a flexible word-order language, Russian.

In conclusion, the present Research Topic will undoubtedly contribute to a better understanding of how neurocognitive systems provide humans with language and will help to further unveil the backstage of our intrinsic communication abilities.

## Author Contributions

OS and YS conceived the paper and the Research Topic. OS, YS, AM, and BM-L contributed to the final version of the manuscript. All authors contributed to the article and approved the submitted version.

## Conflict of Interest

The authors declare that the research was conducted in the absence of any commercial or financial relationships that could be construed as a potential conflict of interest.

